# Adjustment ability and associated factors of outpatients living with cancer

**DOI:** 10.1002/nop2.221

**Published:** 2018-12-14

**Authors:** Keiko Hirokawa, Shizue Suzuki

**Affiliations:** ^1^ Kawasaki University of Medical Welfare Kurashiki Okayama; ^2^ Kobe City College of Nursing Kobe Japan

**Keywords:** adjustment ability, factor, nurses nursing, oncology nursing

## Abstract

**Aims and objectives:**

To clarify the adjustment ability of outpatients with cancer, associated factors and the relationship between adjustment ability and associated factors.

**Design:**

Quantitative study.

**Methods:**

Anonymous self‐reported questionnaire (adjustment ability scale of outpatients with cancer) responses, patient background information and possible associated factors were collected from 369 cancer outpatients.

**Results:**

The mean ± SD adjustment ability score was 110.5 ± 27.2. Several factors were associated with adjustment ability. The Functional Assessment of Cancer Therapy‐General Scale, used to measure quality of life (QOL), was significantly higher in people with an adjustment ability score >95. The factor that was most related to the adjustment ability score was how people think about asking for support from others. The adjustment ability was higher among people who thought, “I should be supported by others for the things I cannot do by myself”.

## 1 INTRODUCTION

The estimated total number of patients with cancer in Japan is more than 300,000 (Statistics & Information Department, Minister’s Secretariat, Ministry of Health, Labour & Welfare, 2014). Among these people, the percentage of outpatients has been increasing and account for more than 50% of the total patients with cancer in statistical research after 2008.

It was reported that outpatients with cancer (hereinafter referred to as patients with cancer) have physical, social, economic and mental/psychological burdens (Horii, Kobayashi, & Suzuki, 2009; Naka, Oishi, & Onishi, 2007; Narui et al., 2004; Yoneda, Fukuda, Yada, & Kakikawa, 2002). Most patients with cancer who have a risk of recurrence or metastasis require long‐term complicated disease management adjustments according to changes in symptoms, conditions or treatments. It is important for these people to develop the ability to continually adjust their life according to these changes for a longer period of time. Patients with cancer live at home, in contrast to inpatients, so it is difficult for them to immediately consult with nurses or ask nurses for support. These observations point to the need for strategies to empower patients with cancer to apply their life adjustment ability in the absence of support.

Most previous studies that clarified factors associated with the life of patients with cancer focused on their quality of life (QOL). They revealed an association between QOL and symptoms (Narisawa, Sato, Kashiwagura, & Sato, 2014), fatigue and self‐efficacy (Mitsuki, Mouri, Sugaya, & Matsuda, 2011). Fujizuka, Ito, Awatsu, Abo, and Noto (2016) focused on the self‐care ability of patients with cancer receiving outpatient chemotherapy and demonstrated that factors such as being older than 65 years, having a spouse, being in contact with fellow people and seeking the cause of health or disease in people themselves or in their family promote self‐care abilities. However, to date, no study has focused on the life adjustment ability of patients with cancer or clarified the details about its associated factors.

Bandura (1996) noted that we behave according to the situation facing us as if we have no self‐regulation ability despite constant changes that may affect us, similar to a weather vane in the wind. On the other hand, Pajares (2008) reported that the capabilities to symbolize, plan alternative strategies, learn through vicarious experience, self‐regulate and self‐reflect provide humans with the cognitive means by which they are influential in determining their own destiny. In other words, we have an influence on our destiny by using our adjustment ability. Although cancer is now considered to be a chronic disease due to advances in medical treatments, it is still associated with death. Patients with cancer think about death, but they can recognize that they can control their destiny by adjusting their entire life. Thus, strengthening people adjustment ability according to changes in their life after developing cancer and providing support to enable continual development of their adjustment ability are considered to be important. Therefore, we conducted this study to clarify patients with cancer adjustment ability, associated factors and the relationship between adjustment ability and associated factors.

## 2 METHODS

### 2.1 Study design

This was a descriptive correlational study using a self‐reported questionnaire.

### 2.2 Study participants

We included patients with cancer 20–70 years of age who were diagnosed with cancer and visiting an outpatient department for follow‐up or continuous treatments, who completed their primary treatments and had been discharged after the initial treatment more than 6 months previously.

### 2.3 Data collection

We asked 15 medical institutions located in western Japan for their cooperation in distributing the questionnaires: linked regional core centres for the treatment of cancer and prefectural‐designated hospitals for the treatment of cancer and general hospitals. Questionnaires with a reply envelope were distributed to study participants who consented. These questionnaires were collected by mail.

### 2.4 Description of questionnaire

#### 2.4.1 Cover sheet

We asked study participants to include patient background information: age, sex, working status (e.g. employed or unemployed, or working pattern), site of cancer, duration from diagnosis of cancer, hospitalization associated with cancer, number of hospitalizations, longest hospitalization period, main purpose of hospitalization, shortest interval between hospital visits, longest interval between hospital visits, purpose of outpatient visit and previous disease other than cancer or injury that adversely affected the patient's daily life for more than 1 month.

#### 2.4.2 Adjustment ability scale of outpatients with cancer

We used the Adjustment Ability Scale of Outpatients with Cancer (hereinafter referred to as “adjustment ability scale”) with validated internal consistency, stability and constant discriminant validity developed by Hirokawa and Suzuki (2018). It is a five‐point scale (from “Very much true” ‐ “Not true at all”), where a higher total score for each item reflects a higher adjustment ability.

#### 2.4.3 Possible factors associated with adjustment ability

It has been shown that “Presence of support for people”, “Physical conditions” and “Recognition of role” are associated with the adjustment ability of patients with cancer (Hirokawa, 2016). We asked the study participants to respond about the “Person who provides daily support”, “Person who provides support when depressed”, “Difficulty due to cancer and treatment (consisting of 12 items including diet, bowel movements and urination)” and “Patient's role in daily life.” We also asked the study participants how they think about their supporter and the participants’ roles based on a five‐point scale (from “Very much true” to “Not true at all”) through the following questions: “I should be supported by others for the things I cannot do by myself” and “I think have a role in a job or in my family that no one else can do”.

#### 2.4.4 Functional assessment of cancer therapy‐general version 4, Japanese version

We used the Functional Assessment of Cancer Therapy‐General (FACT‐G), Japanese version, to assess the QOL of patients with cancer. FACT‐G is a self‐reported questionnaire developed in 1993 by Cella et al. in the United States to measure the QOL of people with cancer. Its reliability and validity have already been demonstrated. It is composed of four subscales with a total of 27 items: physical (seven items), social/familial (seven items), psychological (six items) and functional (seven items). People rate each item on a scale from 1 to 5 (from “Not true at all” to “Very much true”). Scores for all items are added, and a higher score reflects a higher QOL. The Japanese version was developed by Fumimoto et al. (2001), and its reliability and validity have been demonstrated (Shimotsuma & Eguchi, 2001).

### 2.5 Analytical methods

#### 2.5.1 Exploratory analysis of factors associated with adjustment ability

The relationship between responses for items considered to be associated with patients with cancer adjustment ability and adjustment ability scores was analyzed using the Mann–Whitney *U* test or Kruskal–Wallis test.

#### 2.5.2 Relationship between adjustment ability and QOL

We classified the adjustment ability into several different low and high groups. The relationship between these scores and FACT‐G scores was analysed using the Mann–Whitney *U* test.

#### 2.5.3 Factors that influence higher adjustment ability

A path coefficient was calculated for the higher adjustment ability group, with associated factors as the explanatory variable and the adjustment ability score as the objective variable. We used SPSS22.0J (IBM SPSS Amos Authorized User version 21) for the analysis.

### 2.6 Ethical considerations

Before the study began, it was approved by our affiliated institution and the ethics committees of the participating institutions.

We provided study participants with oral and written explanations that their participation in this study was entirely voluntary and not associated with any medical institutions and that anyone could refuse to participate in the study without any loss of benefits in future treatments. We provided anonymous questionnaires only to people who consented to participate in the study, and the questionnaires were collected by mail. Final study participation consent was considered to be obtained when a questionnaire was returned. Completed questionnaires were handled with care to protect personally identifiable information.

## 3 RESULTS

A total of 750 questionnaire forms were sent to 15 institutions, and a total of 409 participants responded (response rate: 54.5%). We excluded 40 responses with a blank answer field in more than 10% of the total number of question items, for a total of 369 valid responses (valid response rate: 49.2%). A mean score was used for 33 blank question items among the valid responses.

### 3.1 Participant characteristics

The study participants included 262 women (71.0%) and 107 men (29.0%) with an age range of 25–69 years (mean: 56.7 years) (Table 1). The most common age group was the 60s (176, 47.7%), followed by the 50s (105, 28.5%). Cancer locations included the breast in 186 participants (50.4%), the gastrointestinal tract in 88 participants (23.8%: oesophagus, stomach and bowel) and a respiratory organ in 70 participants (19.0%: lung and trachea). A total of 301 (81.6%) participants had cancer at one site and 67 (18.2%) had cancer at two or more sites. The duration from the first diagnosis to the survey ranged from 6 months to 43 years and 10 months (mean duration: 4.5 years). The most common duration was 1 to 2 years in 68 participants (18.4%), followed by 6 months to 1 year in 55 (14.9%). More than half of the total participants had been diagnosed with cancer for 6 months to 4 years (221, 59.9%). With respect to difficulties with physical functioning and daily life due to cancer and treatment, 328 participants (88.9%) responded that they had difficulties, whereas 40 participants (10.8%) said that they had no difficulties. The most frequently selected difficulty among multiple answers was physical activity in 236 participants (64.0%), followed by diet in 228 (61.8%).

**Table 1:** Participant characteristics

	Number of people (%) *N* = 369
Sex	
Female	262 (71.0)
Male	107 (29.0)
Age group	
20s	1 (0.3)
30s	19 (5.1)
40s	68 (18.4)
50s	105 (28.5)
60s	176 (47.7)
Mean (± *SD*)	56.6 ± 9.33
Range	25–69
Role in daily life	
Yes (multiple answers allowed)	280 (75.9)
Childcare	38 (10.3)
General housework	268 (72.6)
Caregiving	26 (7.0)
Other	27 (7.3)
Only 1 role	204 (55.3)
≥2 roles	76 (20.6)
No	86 (23.3)
No response	3 (0.8)
Mental supporter	
Yes (multiple answers allowed)	350 (94.9)
Family	320 (86.7)
Friend	198 (53.7)
Neighbour	14 (3.8)
Professional	55 (14.9)
Colleague	45 (12.2)
Other	15 (4.1)
Only 1 type	146 (39.6)
≥2 types	204 (55.3)
No	11 (3.0)
No response	8 (2.2)
Site of cancer (multiple answers allowed)	
Respiratory organ (lung and trachea)	70 (19.0)
Gastrointestinal tract (oesophagus, stomach and bowel)	88 (23.8)
Liver, gallbladder, bile duct and pancreas	32 (8.7)
Urinary organ (bladder, urinary duct, etc.)	11 (3.0)
Prostate	7 (1.9)
Breast	186 (50.4)
Female genital organs (uterus, ovary, etc.)	19 (5.1)
Blood	2 (0.5)
Other	38 (10.3)
Only 1 site	301 (81.6)
≥2 sites	67 (18.2)
No response	1 (1.8)
Period from diagnosis	
6 months to 1 year	55 (14.9)
1–2 years	68 (18.4)
2–3 years	50 (13.6)
3–4 years	48 (13.0)
4–5 years	28 (7.6)
5–6 years	33 (8.9)
6–7 years	20 (5.4)
7–8 years	10 (2.7)
8–9 years	12 (3.3)
9–10 years	8 (2.2)
10–20 years	29 (7.9)
>20 years	8 (2.2)
Mean	4 years and 6 months ±4 years and 11 months
Range	6 months to 43 years and 10 months
Hospitalization	
Yes	350 (94.9)
No	14 (3.8)
No response	5 (1.4)
Number	
1	133 (36.0)
2–5	177 (48.0)
6–10	25 (6.8)
>10	15 (4.1)
Longest period	
≤1 week	89 (24.1)
≤2 weeks	101 (27.4)
≤3 weeks	50 (13.6)
≤1 month	40 (10.8)
≤2 months	50 (13.6)
3–6 months	17 (4.6)
>6 months	3 (0.8)
No response	5 (1.4)
Purpose (multiple answers allowed)	
Surgery	305 (82.7)
Anticancer drug treatment	155 (42.0)
Radiotherapy	40 (10.8)
Hormone treatment	4 (1.1)
Immunotherapy	3 (0.8)
Examination	65 (17.6)
Other	23 (6.2)
No response	3 (0.8)
Hospital visit	
Shortest visit interval	
Every day	92 (24.9)
Once 1–3 days	18 (4.9)
Once 4–7 days	127 (34.4)
Once 10 days to 2 weeks	56 (15.2)
Once 3 weeks to 1 month	59 (16.0)
Once 2–3 months	11 (3.0)
Other	1 (0.3)
No response	5 (1.4)
Longest visit interval	
Once a week	7 (1.9)
Once every 2 weeks	42 (11.4)
Once every 3 weeks to 1 month	156 (42.3)
Once every 2–3 months	116 (31.4)
Once every 4–6 months	33 (8.9)
Once a year	7 (1.9)
Other	1 (0.3)
No response	7 (1.9)
Purpose (multiple answers allowed)	
Follow‐up	267 (72.4)
Outpatient treatment	266 (72.1)
Prescription	191 (51.8)
Examination	300 (81.3)
Other	5 (1.4)
No response	2 (0.5)
Difficulties with physical and daily activities due to disease or treatment	
Yes (multiple answers allowed)	328 (88.9)
Diet	228 (61.8)
Bowel movements and urination	180 (48.8)
Physical activity	236 (64.0)
Retaining posture	133 (36.0)
Sleep	203 (55.0)
Bathing	130 (35.2)
Change and selection of clothes	121 (32.8)
Communication	92 (24.9)
Sex	120 (32.5)
Communication with others	143 (38.8)
Financial situation	186 (50.4)
Hobby	131 (35.5)
Only 1	36 (9.8)
2–5	133 (36.0)
6–9	97 (26.3)
≥10	62 (16.8)
No	40 (10.8)
No response (total number)	31 (8.4)

We compared breast patients with cancer (50.4%) with other patients with cancer to clarify each patient characteristic. The Mann–Whitney *U* test revealed a significantly lower age (*p* < 0.001, *p* < 0.05), significantly longer duration from the first diagnosis (*p* = 0.003), significantly more difficulties (*p* = 0.010) and a significantly greater number of types of people providing mental support (*p* = 0.007) in breast patients with cancer than in other patients with cancer. Conversely, a significantly larger number of hospitalizations were observed in other patients with cancer than in breast patients with cancer (*p* < 0.001, *p* < 0.05). The value of chi‐square was 152.158 (*p* = 0.000, *p* < 0.001) for sex, indicating that the proportion of women was significantly higher in breast patients with cancer, while that of men was significantly higher in other patients with cancer. A significant difference was also observed in the recognition of a role in daily life (*χ* ^2^ = 73.246, *p* < 0.001, *p* < 0.001) and hospital visit experience for treatment (*χ* ^2^ = 10.662, *p* = 0.001) between breast patients with cancer and other patients with cancer. With respect to how they think about asking for support from others, a significant difference was observed in the “Somewhat true” and “Very much true” responses to “I should be supported by others for the things I cannot do by myself” between these patient groups (*χ* ^2^ = 13.224, *p* = 0.01). There was also a significant difference between these groups in the shortest interval between hospital visits (*χ* ^2^ = 73.410, *p* < 0.001, *p* < 0.001) and the longest interval between hospital visits (*χ* ^2^ = 32.308, *p* < 0.001, *p* < 0.001). On the other hand, no significant difference was observed in the number of cancer sites (*p* = 0.212), the number of types of people who provide mental support for people (*p* = 0.527), working status (*p* = 0.768) and previous disease other than cancer or injury that adversely affected a patient's daily life for more than one month (*p* = 0.203).

### 3.2 Adjustment ability

The mean (±*SD*) adjustment ability score was 110.5 (SD  27.2; range: 45–185), with a median score of 109.0. The Kolmogorov–Smirnov test for the adjustment ability score revealed a normal distribution (*p* = 0.200).

### 3.3 Exploratory analysis of factors associated with adjustment ability

We performed a statistical exploratory analysis of the relationship between possible factors associated with the adjustment ability of patients with cancer and their adjustment ability score. The analysis demonstrated several factors associated with adjustment ability: sex, age group, interval between hospital visits, purpose of outpatient visit, people who provide mental support for people, difficulties associated with disease or treatment and period from diagnosis. Specifically, people with a greater adjustment ability had one of the following characteristics: female sex, in their 40s, the shortest time between hospital visits at once every 4–7 days, the longest time between hospital visits at once every week to every month, previous experience with hospital visits for treatment, having three people providing mental support, difficulties associated with disease or treatment and those who satisfy three conditions one or more difficulties with physical and daily activities due to disease or treatment, the shortest interval between hospital visits at approximately every day to once a week and a period from diagnosis of 6 months to 3 years.

### 3.4 Relationship between adjustment ability and QOL

We conducted a statistical analysis for the relationship between the FACT‐G score (the existing scale that assesses QOL in patients with cancer) and the adjustment ability score. We classified the adjustment ability scores into a low (score lower than 100:133 people) and a high group (score 100 or higher: 236 people) and performed the Mann–Whitney *U* test for their association with the FACT‐G score. The test revealed a significant difference (*p* = 0.013): The FACT‐G score was significantly higher in the high adjustment ability group than the low group, indicating a higher QOL in the former group.

We then adjusted the cut‐off score for the low and high groups at an adjustment ability score lower (109 people) or higher (260 people) than 95, respectively, and conducted the same statistical tests as described above. This test revealed a significant difference (*p* = 0.049). Then, we performed another Mann–Whitney *U* test with the low group (adjustment ability score lower than 94) and the high group (score of 94 or higher) and found no significant difference (*p* = 0.060). These findings demonstrated that there was a difference in QOL between people who had an adjustment ability score of 95 or higher and those who had a score lower than 95.

### 3.5 Factors that influence adjustment ability

We identified factors that influence high adjustment ability. Also, we demonstrated the relationship between adjustment ability and factors associated with adjustment ability in 369 study participants.

For 266 people with hospital visit experience for treatment, we performed an analysis with the following explanatory variables: sex, age group, working status, recognition of role, number of types of people who provide daily support or mental support for people, number of cancer sites, period from diagnosis, number of hospitalizations, shortest interval between hospital visits, longest interval between hospital visits, number of difficulties associated with disease or treatment, previous disease other than cancer or injury that adversely affected the patient's physical condition or daily life for more than 1 month and how they think about asking for support from others and the adjustment ability score as the objective variable. This analysis revealed that the factor that influenced the adjustment ability score the most was how people think about asking for support from others (path coefficient: 0.28). It showed that people who thought “I should be supported by others for the things I cannot do by myself” had a higher adjustment ability. The next most influential factors included the period from diagnosis (path coefficient: 0.21), the number of types of people who provide mental support for people (path coefficient: 0.16) and the number of difficulties associated with disease or treatment (path coefficient: 0.14). The path coefficient for the influence of age group, working status and longest time between hospital visits on the adjustment ability score was below zero.

We performed a similar analysis for 266 people with one or more difficulties in physical and daily activities due to disease or treatment, a shortest time between hospital visits of approximately every day to once a week and a period from diagnosis of 6 months to 3 years, as described above. Explanatory variables included sex, age group, working status, recognition of role, number of types of people providing daily support or mental support for people, number of cancer sites, period from diagnosis, number of hospitalizations, shortest interval between hospital visits, longest interval between hospital visits, hospital visit experience for treatment, number of difficulties associated with disease or treatment, previous disease other than cancer or injury that adversely affected the patient's physical condition or daily life for more than 1 month and how the patient thinks about asking for support from others (support from others). The objective variable was the adjustment ability score. This analysis revealed that the most influential factors on adjustment ability score included the number of types of people who provide mental support for people (path coefficient: 0.23) and the shortest time between hospital visits (path coefficient: 0.23). The next most influential factors included how a patient thinks about asking for support from others (path coefficient: 0.18), working status (path coefficient: 0.16) and period from diagnosis (path coefficient: 0.13). The path coefficient for the effect of age group, number of types of people providing daily support, number of cancer sites and the longest time between hospital visits on a patient's adjustment ability score was below zero.

**Figure 1:**
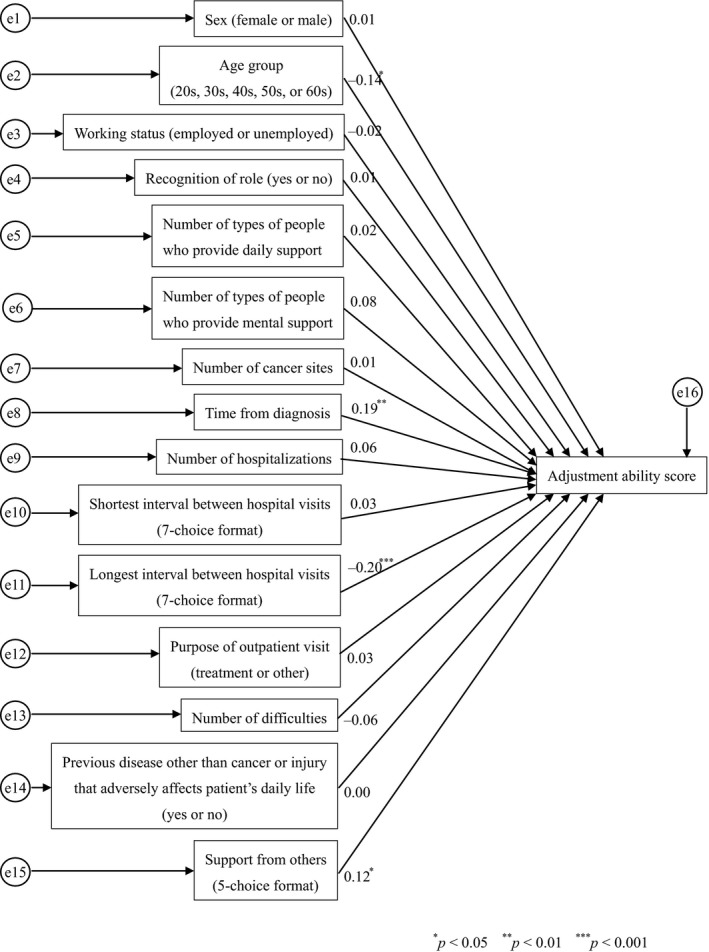
Path diagram of adjustment ability and associated factors in people with an adjustment ability score ≥95

For 260 people with an adjustment ability score of 95 or higher, we performed a similar analysis as described above. The explanatory variables included sex, age group, working status, recognition of role, number of types of people providing daily support or mental support for people, number of cancer sites, period from diagnosis, number of hospitalizations, shortest interval between hospital visits, longest interval between hospital visits, hospital visit experience for treatment, number of difficulties associated with disease or treatment, previous disease other than cancer or injury that adversely affected a patient's physical condition or daily life for more than 1 month and how a patient thinks about asking for support from others (support from others). The objective variable was the adjustment ability score (Figure 1). This analysis revealed that the factor that had the most influence on the adjustment ability score was the longest interval between hospital visits (path coefficient: −0.20). People with a shorter longest time between hospital visits had a higher adjustment ability. The next most influential factors included the period from diagnosis (path coefficient: 0.19), age group (path coefficient: −0.14) and how a patient thinks about asking for support from others (path coefficient: 0.12). The path coefficient for the effect of age group, working status, longest interval between hospital visits, hospital visit experience for treatment and number of difficulties associated with disease or treatment on a patient's adjustment ability score was below zero.

**Figure 2:**
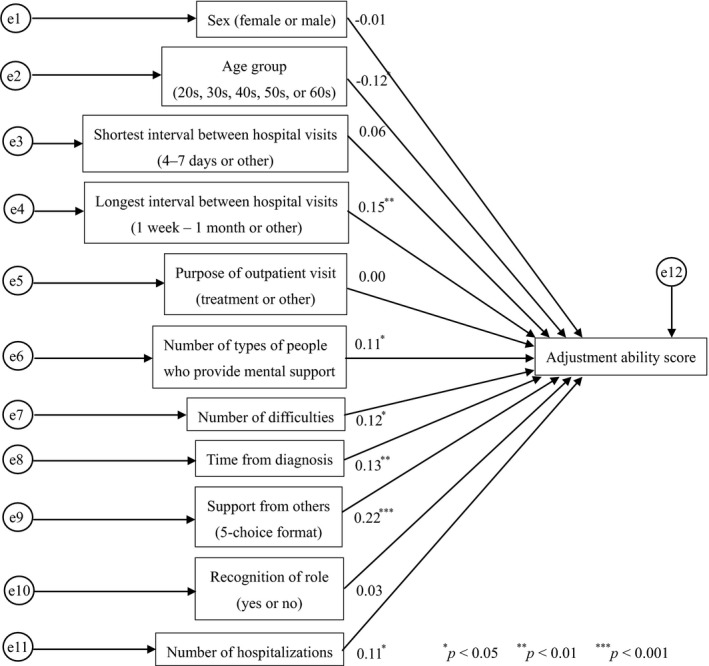
Path diagram of adjustment ability and associated factors

We used nine factors that were shown to be associated with adjustment ability as explanatory variables, including sex, age group, shortest interval between hospital visits, longest interval between hospital visits, purpose of outpatient visits, number of types of people who provide mental support for people, number of difficulties associated with disease or treatment, period from diagnosis and how a patient thinks about asking for support from others (support from others) and two additional factors (recognition of role and number of hospitalizations) in an analysis similar to the one described above (Figure 2). This analysis revealed that the factor that had the most influence on a patient's adjustment ability score was how the patient thinks about asking for support from others (path coefficient: 0.22). It also showed that people who thought, “I should be supported by others for the things I cannot do by myself” had a higher adjustment ability． The next most influential factors included the longest interval between hospital visits (path coefficient: 0.15) and the period from diagnosis (path coefficient: 0.13). The path coefficient for the effect of age group on a patient's adjustment ability score was below zero.

## 4 DISCUSSION

### 4.1 Adjustment ability based on associated factors

The multivariate and univariate analyses for factors associated with adjustment ability suggested that people who had a higher adjustment ability were in their 40s with the longest interval between hospital visits being once every week to every month, had three people providing mental support, difficulties associated with disease or treatment, many hospitalizations, one or more difficulties in physical and daily activities due to disease or treatment, the shortest interval between hospital visits being approximately every day to once a week and a period from diagnosis of 6 months to 3 years. Based on this finding, we believe that people can use their adjustment ability in difficult situations (e.g. a shorter time between hospital visits or difficulties with physical and daily activities). The number of hospitalizations was associated with people adjustment ability, possibly due to the necessity of adjusting the people roles in their jobs or families during hospitalizations. The most influential factor on adjustment ability in people who had an ability score of 95 or higher was the longest interval between hospital visits (path coefficient: −0.20), and it was shown that people with the longest times between hospital visits had a lower adjustment ability. The longer time between hospital visits may reflect the fact that a patient did not need to adjust to the visit and, therefore, did not need to use this ability. Therefore, it is thought that a patient's adjustment ability is not required when adjustment is not required or only small adjustments are needed.

The analysis of factors associated with a higher adjustment ability showed that how people think about asking for support from others affects the adjustment ability of people with an ability score of 95 or higher. In other words, “I should be supported by others for the things I cannot do by myself” had a higher adjustment ability score. In people with one or more difficulties in physical and daily activities due to disease or treatment, a shortest time between hospital visits of approximately every day to once a week and a period from diagnosis of 6 months to 3 years, the most influential factor on adjustment ability was the number of types of people who provide mental support. In people who had prior experience with hospital visits for treatment, the path coefficient for the number of types of persons who provide mental support for people (0.16) was higher than that of the number of difficulties associated with disease or treatment (0.14). This suggests that people who have a greater number of types of people who provide mental support show a higher adjustment ability, even though they have many difficulties.

### 4.2 Meaning of increased adjustment ability

Kondo, Shimizu, Watanabe, Fukuda, and Oishi (2004) reported that cancer survivors can be self‐reliant by regaining and improving their ability to cope with issues within themselves, being autonomous and asking others for support. It is important to focus on a cancer patient's own abilities. Patients with cancer can change their life on a moment‐to‐moment basis according to the situation they face or continuously by improving their adjustment ability so they can cope with various problems or control difficult situations in physical and daily activities.

Our results showed a significantly higher QOL in the high adjustment ability score group (95 or higher) than in the low score group (lower than 95; *p* = 0.049). This study demonstrated an association between higher adjustment ability and higher QOL (e.g. physical and emotional security and living by people own values), suggesting the importance of increasing adjustment ability in patients with cancer.

### 4.3 Application to nursing practice to improve the adjustment ability of patients with cancer

#### 4.3.1 Changing people perception of support from others

The path coefficient of how a patient thinks about asking for support from others to the adjustment ability of patients with cancer was 0.22. People who thought, “I should be supported by others for the things I cannot do by myself” had a higher adjustment ability, and this was the most influential factor on their adjustment ability.

A literature review revealed that patients with cancer have physical, social, economic and mental/psychological burdens and have to change their whole life because it is difficult for them to live in the same way as they lived before cancer onset. They must manage various aspects of their life, and there may be some things they cannot cope with on their own. They may give up trying to manage these aspects if they do not think that they have adequate support from others for the things they cannot do by themselves. But they may be hesitant about requesting support from others because they had lived independently and played many roles in their family or society before cancer onset. Nakazawa, Kanda, Kyota, and Honda (2014) noted that there is an association between the severity of a disorder and the degree of dependence of people and that people with more severe symptoms can do fewer things for themselves and have reduced independence. They also reported that these people tend to deny and devalue themselves．It is thought that there is a close association between receiving support from others and the independence of patients with cancer. It has also been demonstrated that working patients with cancer who underwent their first chemotherapy received support from others with hesitation (Tanaka & Tanaka, 2012). Maeda, Oishi, and Hayama (2012) reported that support for people, such as those providing direct physical care, mental care or medical personnel providing specialized knowledge, affected the whole process of reorganizing a patient's life in rectal patients with cancer who had received total pelvic exenteration.

Therefore, we need to aid patients with cancer in receiving support from others without feeling devalued. It is important for patients with cancer to realize that receiving support from others does not mean dependence. We must support them to receive proper assistance from others with full respect for their independence to improve their adjustment ability. First, we should assess how patients with cancer react to what they cannot do. We need to understand their way of thinking about asking for support from others because it reflects their individual value and the way they live. Also, correct recognition of support provided by others (i.e. temporary and is not a support for all things) is likely to be necessary.

#### 4.3.2 Support to overcome difficulties associated with disease or treatment

The path coefficient of the number of difficulties associated with disease or treatment to the adjustment ability of patients with cancer was 0.12. There is a positive association between the number of difficulties a patient faces and their adjustment ability, suggesting that a higher adjustment ability is required for people facing more difficulties and that the ability can be improved if patients with cancer cope with these difficulties. Therefore, the adjustment ability can be improved if patients with cancer cope appropriately with difficulties in physical and daily activities.

A shorter interval between hospital visits, symptoms associated with disease progression and adverse events are a heavy physical burden on patients with cancer with decreased strength. Also, it is not easy for discharged patients with cancer to secure time for hospital visits while still playing the roles they have to take on after being discharged. Difficulties associated with disease or treatment can increase the necessity for people to control their lives. Physical reactions to cancer differ between people, and it is necessary to recognize small physical changes and understand each patient's condition. It is reported that recognition and understanding allow patients with cancer to find their own ways to cope with difficulties (Kosaka & Majima, 2011). Patients with cancer are likely to think, carry out and evaluate the way they cope with difficulties using the “Ability to think” and “Ability to understand and control people themselves” and being worried about their physical reactions to cancer or finding a new way to control their life. It can be important for us to observe these physical reactions and to evaluate people actions to manage difficulties associated with disease or treatment.

#### 4.3.3 Support to increase the number of types of people who provide mental support for people

The path coefficient of the number of types of people who provide mental support for people to the adjustment ability of patients with cancer was 0.11. The path coefficient of the number of difficulties associated with disease or treatment to the adjustment ability was similar at 0.12. These two factors have a similar impact on a patient's adjustment ability. People who provide mental support for people will promote improved adjustment ability in those who can use the ability despite difficulties. On the other hand, those who cannot use the ability due to difficulties can improve their adjustment ability if they have several people providing mental support. The feeling of being supported by others helps patients with cancer to continue chemotherapy (Nishikawa, Funahashi, & Kuroda, 2015). It is thought that the presence of those who provide mental support helps patients with cancer improve their physical functions and daily activities instead of directly helping these people to manage their lives.

In this modern world with an ageing population and fewer births, it is difficult to receive sufficient mental support from family. Also, our finding that people who have a greater number of types of people who provide mental support had a higher adjustment ability suggests that mental support from a patient's family alone is not enough. Therefore, patients with cancer should be mentally supported by a consultation service and a network of people including their colleagues and fellow people, as well as medical personnel.

## 5 STUDY LIMITATION AND FUTURE ISSUES

A limitation of the study was that people with a lower adjustment ability included those who cannot use the ability despite the necessity for adjustment and those who use the ability despite not needing adjustment.

Possible results of people using an adjustment ability include an improved QOL, completion of outpatient treatment and continuous hospital visits. We should aim to clarify the association between using adjustment ability and the completion of outpatient treatment or continuous hospital visits in the future. Also, a nursing support model focusing on the process for improving the adjustment ability of patients with cancer should be developed to promote this ability.

## 6 CONCLUSION

This study revealed the following factors associated with the adjustment ability of patients with cancer: sex, age group, interval between hospital visits, purpose of outpatient visit, persons who provide mental support for people, difficulties associated with disease or treatment and period from diagnosis. People with a higher adjustment ability had a better QOL. It was also shown that people who thought, “I should be supported by others for the things I cannot do by myself” had a higher adjustment ability.

## CONFLICT OF INTERESTS

The authors have no funding or conflict of interests to disclose.

## AUTHOR CONTRIBUTIONS

Study design: KH and SS; Data collection and analysis: KH; Manuscript preparation: KH and SS.
